# Supplementary material for: miR2119, a Novel Transcriptional Regulator, Plays a Positive Role in Woody Plant Drought Tolerance by Mediating the Degradation of the CkBI-1 Gene Associated with Apoptosis
Source: Int J Mol Sci. 2022 Jun 4;23(11):6306. doi: 10.3390/ijms23116306 (PMC9181555; doi:10.3390/ijms23116306)
Supplement: Supplementary file 1 [file ijms-23-06306-s001.zip › ijms-1704502-supplementary.pdf]

miR2119, a Novel Transcriptional Regulator, Plays a Positive Role in Woody Plant Drought Tolerance by Mediating the Degradation of the *CkBI-1* Gene Associated with Apoptosis

Fu-Rong Liu [1], Pu-Zhi Zhang [1], Jia-Yang Li [2], Tian-Xin Zhang [1], Li-Fang Xie [1] and Chun-Mei Gong [2,*]

[1] College of Life Sciences, Northwest A&F University, Yangling, Shaanxi Province,China; 2809694906@qq.com(F.-R.L.); 1299346584@qq.com(P.-Z.Z.); tianxinzhang@nwafu.edu.cn(T.-X.Z.);1347989419@qq.com(L.-F.X.)

[2] College of Horticulture, Northwest A&F University, Yangling, Shaanxi Province,China; jiayang@nwafu.edu.cn(J.-Y.L.)

* Correspondence: gcm228@nwafu.edu.cn(C.-M.G.)

Table S1 Related gene cloning primer sequences of *C. korshinskii*.

Primer name	Primer sequence(5′-3′)
miR319-F	CATGCCATGGCAAACACACGCTCGGACG
miR319-R	GAAGATCTGGGATGCCTTAAATAAAGATAAACCC
CkmiR2119-I	GATCAAAGGGAGGTGTGGAGTAGTCTCTCTTTTGTATTCC
CkmiR2119-II	GACTACTCCACACCTCCCTTTGATCAAAGAGAATCAATGA
CkmiR2119-III	GACTCCTCCACACCTGCCTTTGTTCACAGGTCGTGATATG
CkmiR2119-IV	GAACAAAGGCAGGTGTGGAGGAGTCTACATATATATTCCT
CkBI-1-F	CATGCCATGGACGCGTTTACATCGTTC
CkBI-1-R	TGAAGATCTGGATCTTTCCTCTTCTTCTTCCT

Table S2 qRT-PCR primer sequences of *C. korshinskii*.

Primer name	Primer sequence(5′-3′)
RT-CkmiR2119-F	GCGGCGGTCAAAGGGAGGTGTG
RT-CkmiR2119-R	ATCCAGTGCAGGGTCCGAGG
RT-CkBI-1-F	CGATACTCTCAAGAACTTCCGTCAG
RT-CkBI-1-R	CATGGTTCCAAGGGACGCTAC
u6-F	GGAACGATACAGAAGATTAGCA
u6-R	GTGCAGGGTCCGAGGT
CkUB-F	GACTTTGACCGGGAAGACCA
CkUB -R	CACCACGAAGACGGAGCACA

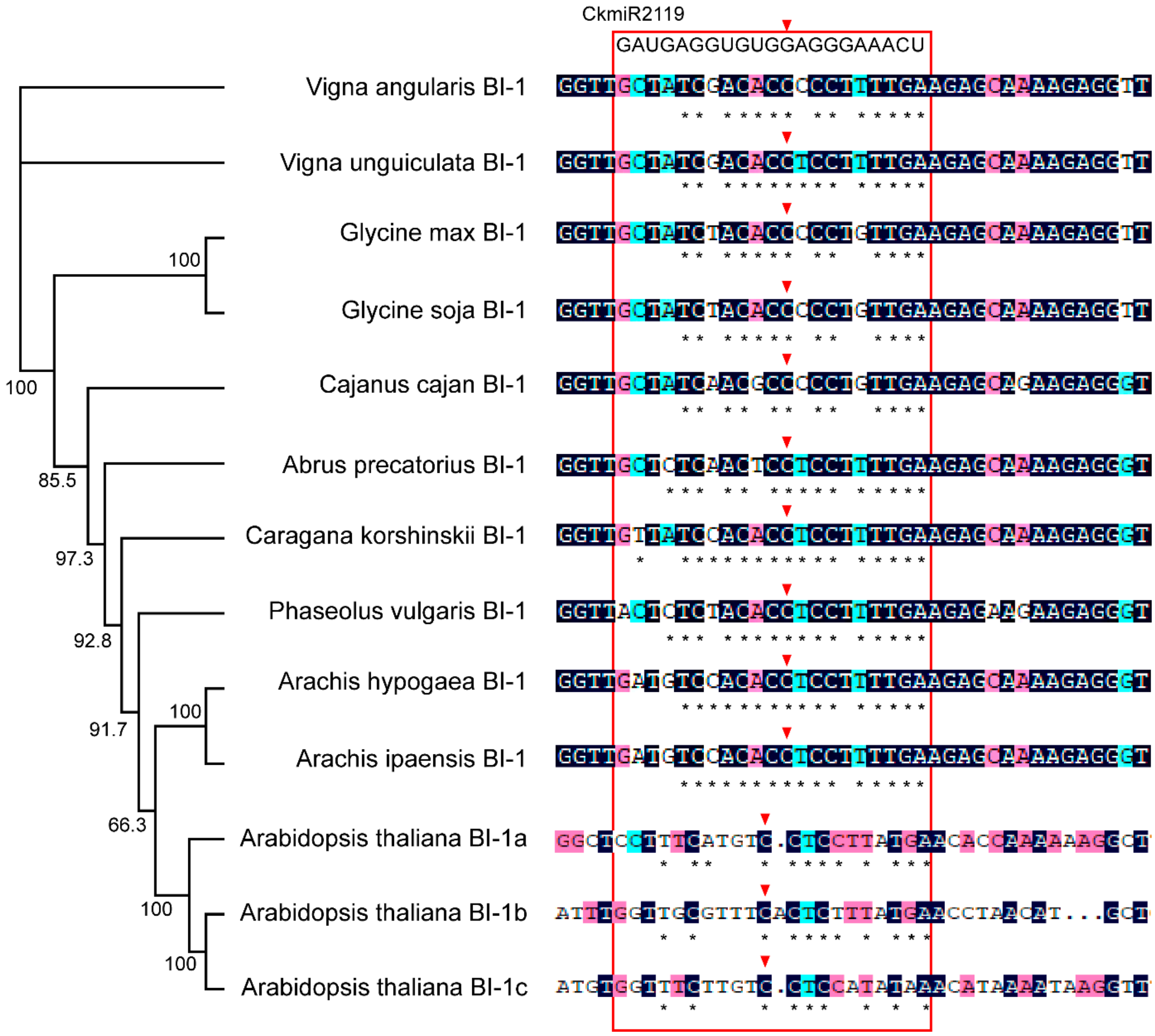

Figure S1. Phylogenetic tree analysis and multiple sequence alignment of *BI-1* in *C. korshinskii*, *Glycine max*, *Glycine soja*, *Cajanus cajan*, *Vigna angularis*, *Abrus precatorius*, *Phaseolus vulgaris*, *Arachis hypogaea*, *Arachis ipaensis*, *Arabidopsis thaliana* and *Vigna unguiculata*. The phylogenetic tree was constructed using the full-length CDS sequences of the BI-1 gene of the above species. Bootstrap support (1000 repetitions) is shown for each node. * indicates that this base can pair with CkmiR2119.

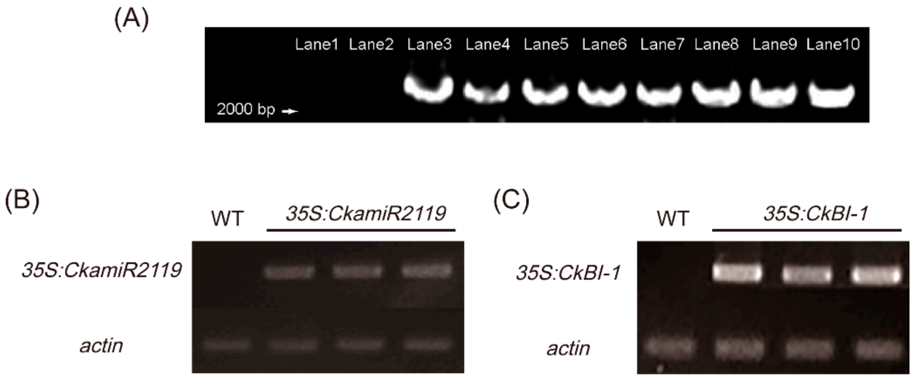

Figure S2. Screening of *CkamiR2119* and *CkBI-1* transgenic tobacco plants. (A) GUS gene detection of T_0 transgenic plants. Lane 1 was water, Lane 2 was wild control plant, Lane 3 was vector, Lane 4 - Lane 6 were *CkamiR2119* transgenic tobacco plants, Lane 7 - Lane 8 were *CkBI-1* transgenic tobacco plants, Lane 9 - Lane 10 were vector transgenic tobacco plants. (B) qRT-PCR identification of T_3 generation *CkamiR2119* transgenic plants. (C) qRT-PCR identification of T_3 generation *CkBI-1* transgenic plants.
